# A silent presentation of massive staghorn calculi

**DOI:** 10.1590/2175-8239-JBN-2023-0072en

**Published:** 2023-11-03

**Authors:** Pedro Lisboa-Gonçalves, Adriana Santos, Teresa Pina-Vaz

**Affiliations:** 1 Centro Hospitalar Universitário São João, Porto, Portugal.; 2 Universidade do Porto, Faculdade de Medicina, Departamento de Medicina, Porto, Portugal.; 3 Universidade do Porto, Faculdade de Medicina, Departamento de Cirurgia e Fisiologia, Porto, Portugal.

## Case Report

**Figure 1.**
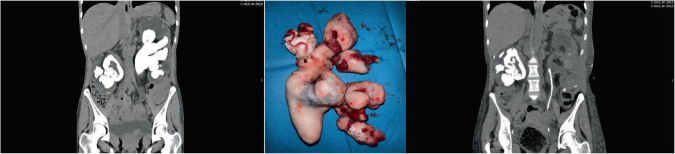
Removal of a massive staghorn calculi by left anatrophic nephrolithotomy and pyelolithotomy. A: Pre-operative CT scan showing bilateral staghorn calculi and dimensional asymmetry between the kidneys. The right kidney appeared with a lower volume and higher parenchymal rarefaction. B: Removed staghorn calculi. C: CT scan showing complete removal of the calculi from the left kidney.

A 44-year-old woman with history of recurrent urinary tract infections (UTI) but no other comorbidities underwent a CT scan revealing bilateral renal staghorn calculi (Figure 1A) after being referred to our department due to CKD diagnosis.

Blood tests revealed a glomerular filtration rate (GFR) of 25 mL/min/1.73 m^2^ with unremarkable additional metabolic assessment. Her urinary sediment showed pH of 7.5 with leukocyturia. A 24-hour urine collection ruled out increased excretion of calcium, uric acid, or oxalates but revealed proteinuria of 2g.

To preserve the remaining kidney function, the patient underwent left anatrophic nephrolithotomy and pyelolithotomy (Figure 1B), which resulted in complete removal of calculi (Figure 1C). Following the intervention, GFR remained stable at 20 mL/min/1.73 m^2^. Stone analysis confirmed the presence of struvite calculi.

Staghorn calculi are associated with recurrent UTI caused by urease-producing organisms^1^. Treatment is based on removal of kidney stones in conjunction with antibiotics^2^.

Choosing anatrophic nephrolithotomy over alternative, non-invasive procedures has the advantage of addressing complex stone burdens and reducing the risk of residual fragments and the need for multiple interventions^3,4^.
